# Hemostasis during hypospadias surgery via topical application of feracrylum citrate: A randomized prospective study

**DOI:** 10.4103/0971-9261.71746

**Published:** 2010

**Authors:** Brijesh K. Lahoti, Gaurav Aggarwal, Arvind Diwaker, Shashi S. Sharma, Ashok Laddha

**Affiliations:** Department of Surgery, M.G.M Medical College & M.Y.H Group of Hospitals, Indore, Madhya Pradesh - 452 001, India

**Keywords:** Hemostasis, hypospadias surgery, topical feracrylum citrate

## Abstract

**Aim:**

Report of our experience with topical feracrylum citrate to minimize hemorrhage-related complications in pediatric hypospadiac patients.

**Materials and Methods:**

One hundred and fifty consecutive pediatric hypospadiac patients over 3 years (75 in study group and 75 controls - random allocation) were studied. One hundred milliliter of 1% feracrylum citrate solution was used in study cases and equivalent normal saline in controls. The parameters assessed were frequency of cauterizations, intraoperative blood loss, wound edema and postoperative complications.

**Results:**

Average number of cauterizations was 1.55 per patient in study group and 5.7 per patient among controls. Among cases, average number of blood soaked gauge pieces was 3.56 per patient, correlating with average intraoperative blood loss of 17.8 ml. In controls, average blood soaked gauge pieces were 6.2 per patient corresponding to an average blood loss of 31 ml. Postoperative hematoma was seen in 8% cases compared with 18% controls. Wound edema appeared in 13.3% cases and 47% controls. Postoperative complications were higher among controls.

**Conclusions:**

Feracrylum is an effective and safe topical hemostatic agent to minimize significantly diffuse capillary oozing and surface bleeding. It reduced the frequency of cauterization and tissue damage, intraoperative blood loss, postoperative hematoma, wound edema and postoperative complications.

## INTRODUCTION

One of the most common problems during hypospadias surgery is bleeding. A relatively new, external, chemical, hemostatic agent – feracrylum – has revolutionized the field of hemostasis and has become a dependable companion in surgery. Its use has been evaluated in varied surgical and diagnostic procedures; however, ours is probably the index study, correlating its use and the outcomes post-hypospadias surgery. The novelty of our study lies in the very procedure that it is being used i.e. in hypospadias surgery. This surgical intervention is performed in pediatric population, majority of who are infants, wherein every milliliter of blood loss makes a significant difference to the patient, and more so, since this age group is also more prone for infection due to inadequately developed acquired immune responses.

## MATERIALS AND METHODS

This study was conducted in 150 consecutive pediatric hypospadiac patients [6 months to 3 years age], (75 cases and 75 controls - random allocation) admitted in our hospital, who underwent urethroplasty over two years from 2007 to 2009.

Among cases, 100 ml of 1% feracrylum citrate solution was used during surgery to achieve hemostasis, via 10×10 cm, loose knit gauge pieces, each soaked in 2 ml of feracrylum solution, applied for 2–3 min, at the bleeding site, with moderate pressure. In the control group, the same 10×10 cm loose knit gauge pieces were soaked in 2 ml normal saline and also applied for 2–3 minutes, with moderate pressure.

All patients underwent the same hypospadias surgery [Snodgrass urethroplasty], by the same surgeon. All patients were administered the same antibiotics both pre- and post-operatively. All patients were followed up monthly after discharge.

We assessed the frequency of cauterization, blood loss during surgery, wound edema and postoperative complications in both groups – the cases as well as the controls. According to our standardization criteria, the average number of blood-soaked gauge pieces were converted into blood loss [in ml]. {A single gauze piece [10×10 cm, loose knit], was found to absorb 5 ml of blood completely, on topical application over an oozing site, irrespective of the compound that the gauze was soaked in, calculated via the difference in weight before and after application}

## RESULTS

All our patients ranged from 6 months to 3 years of age with an average age of 1 year.

The average number of cauterizations were 1.55 per patient in the cases [range 0–5], while they were 5.7 per patient [range 3–14], among the controls.

Blood loss during surgery was assessed by the number of blood-soaked gauge pieces intraoperative, which were previously soaked in feracrylum (cases) or in normal saline (controls) and then converted into average amount of blood loss during surgery (according to our prior standardization criteria).

Among cases, the average number of blood-soaked gauge pieces was 3.56 per patient [range 2–7] correlating with an average amount of blood loss during surgery of 17.8 ml. In the control group, the average number of blood-soaked gauge pieces was 6.2 per patient [range 4–12] corresponding to an average amount of blood loss of 31 ml. Postoperative hematoma (irrespective of size) was seen in 8% of cases, as compared to 18% of controls. Wound edema appeared in 13.3% cases and 47% of controls. The complications encountered by us postoperatively are depicted in Table 1. At the end of two years, 86% of patients are on follow-up.

**Table 1:** Postoperative complications

Complication	Cases (6) (8%)		Controls (22) (29%)		*P* Value
	No.	%	No.	%
Infection	2	2.7	8	10.6	2.45 (*P*<0.05)
Urethrocutaneous fistula	2	2.7	7	9.3	3.02 (*P*<0.05)
Stricture	1	1.3	2	2.7	1.38 (*P*>0.05)
Meatal Stenosis	1	1.3	3	4	1.74 (*P*>0.05)
Devitalized Skin Flap	-	-	2	2.7	2.73 (*P*<0.05)

## DISCUSSION

Feracrylum solution is a novel topical hemostatic agent, for use in control of oozing in various surgical procedures.[1] It also possesses antimicrobial properties and decreases the postoperative infection. Its mode of action is via activation of thrombin, which subsequently causes conversion of fibrinogen to fibrin and thus clot formation. In addition, on coming in contact with serum proteins, it forms a thin film, thus acting as a mechanical barrier preventing exogenous contamination. Feracrylum has a high molecular weight of 5,00,000–8,00,000 Daltons, thus has no systemic absorption and no adverse effects on the liver, kidney, adrenals, cardiovascular and hemopoietic systems.[2]

The beneficial role of feracrylum in hypospadias surgery has not been studied till date. Ours was the first study to evaluate the hemostatic effect of feracrylum in hypospadias surgeries. In our study only feracrylum was used as the hemostatic agent and no comparison was done with other hemostatic agents, rather we compared the benefits of feracrylum among cases as against controls, in whom feracrylum was not used.

Average number of cauterizations in cases was 1.55 (range 0–5) as compared to controls where average was 5.7 (range 3–14), signifying a substantial reduction in the average number of cauterizations (*P*<0.01). On an average, blood loss during surgery in cases was 17.8 ml (avg. 3.56 gauge pieces) compared to 31 ml (avg. 6.2 gauge pieces) in controls. This signifies 13.2 ml less blood loss in cases than controls (*P*<0.01). Postoperative hematoma was observed in only 8% patients (in cases) while, it was seen among 18% of controls, indicating a significant reduction in hematoma formation postoperatively (*P*<0.01).

Wound edema was observed around the periphery of the wound in only 13.3% cases as compared to controls, where it was found in approximately 47% patients. A study conducted in 2004 by Rao *et al*,[3] found that no edema was observed in 33% of patients after using feracrylum gel. In majority of remaining 66%, mild to moderate edema was present. In our present study, approximately three times reduction in wound edema was found among cases (*P* <0.01).

Serious adverse effects have been reported via the use of various hemostatic agents mainly due to the formation of granulomatous masses at the site of usage.[4–6] Feracrylum, however, in our study, proved contrary.

Feracrylum is superior to povidone iodine for its antimicrobial properties.[7] In addition to its antimicrobial activity, feracrylum reduces the frequency of cauterizations, thereby decreasing the chances of tissue necrosis and post-surgical infection. Infection was found in 2.70% (2 patients) of cases and 10.6% (8 patients) of controls, in our study. This signifies a three times reduction of infection in cases as compared to controls (*P*<0.05).

Urethrocutaneous fistulae (UCF) reported after hypospadias surgery result from failure of healing along the neourethral suture line. This healing is hampered due to the use of cauterization in the subcutaneous plane, as found in hypospadias surgery. A reduction in the frequency of cauterizations decreases tissue necrosis and improves the healing. In addition, its hygroscopic property also aids healing, thus reducing UCF occurrence. In our study, UCF occurred in 2.7% (2 patients) in cases, while 9.3% (7 patients) in controls, thus signifying a five times reduction (*P*<0.05), proving that it is safe to use feracrylum citrate as hemostatic agent in hypospadias surgery.

Meatus can become stenotic by crusting, edema or synechiae. Most likely cause for meatal stenosis is vascular compromise of the urethra at the apex of the meatus. Such compromise may be secondary to an inadequate glans channel that compresses blood flow in the pedicle. Meatal stenosis was observed in 1.3% (1 patient) of cases and 4% (3 patients) of controls, signifying three times reduction in meatal stenosis among study cases. Stricture of the neourethra may occur anywhere along its course. However, most common sites of stricture formation are the meatus and the proximal anastomosis. Stricture was seen in 1.3% (1 patient) of cases and 2.7% (2 patients) of controls, implying a two times reduction in stricture formation in cases than controls. Devitalized skin flaps were only found in controls, not in cases (*P*<0.05).

As can be seen from above, topical feracrylum solution application significantly reduces infection, UCF, meatal stenosis and stricture occurrence, thus proving beyond doubt that its application is advantageous. In the present study, it is shown that feracrylum is an agent which not only controls bleeding but also reduces the frequency of cauterization and tissue damage, blood loss during surgery and postoperative hematoma, wound edema and post-surgical complications significantly.

Studies evaluating the use of this novel hemostat in laparoscopic cholecystectomy[7] as well as gastrointestinal bleeds[8] have been conducted; however, large-scale and multicenter trials are mandatory to establish its efficacy in a wider spectra of procedures.

Feracrylum is thus an effective, safe and dependable topical hemostatic agent. It reduces the frequency of cauterization and tissue damage, blood loss during surgery, postoperative hematoma, wound edema and post-surgical complications. It significantly minimizes diffuse capillary oozing and surface bleeding, and thus obtains a clear field during surgery of hypospadias.
